# Finding gaps in TB notifications: spatial analysis of geographical patterns of TB notifications, associations with TB program efforts and social determinants of TB risk in Bangladesh, Nepal and Pakistan

**DOI:** 10.1186/s12879-020-05207-z

**Published:** 2020-07-10

**Authors:** Margo van Gurp, Ente Rood, Razia Fatima, Pushpraj Joshi, Sharat Chandra Verma, Ahmadul Hasan Khan, Lucie Blok, Christina Mergenthaler, Mirjam Irene Bakker

**Affiliations:** 1 KIT Royal Tropical Instituter, Amsterdam, Netherlands; 2 National TB Control Program, Islamabad, Pakistan; 3 National Tuberculosis Programme, Kathmandu, Nepal; 4 National Tuberculosis Control Program, Dhaka, Bangladesh

**Keywords:** Tuberculosis, Case notification, Spatial analysis, GIS, Access to healthcare

## Abstract

**Background:**

In order to effectively combat Tuberculosis, resources to diagnose and treat TB should be allocated effectively to the areas and population that need them. Although a wealth of subnational data on TB is routinely collected to support local planning, it is often underutilized. Therefore, this study uses spatial analytical techniques and profiling to understand and identify factors underlying spatial variation in TB case notification rates (CNR) in Bangladesh, Nepal and Pakistan for better TB program planning.

**Methods:**

Spatial analytical techniques and profiling was used to identify subnational patterns of TB CNRs at the district level in Bangladesh (*N* = 64, 2015), Nepal (*N* = 75, 2014) and Pakistan (*N* = 142, 2015). A multivariable linear regression analysis was performed to assess the association between subnational CNR and demographic and health indicators associated with TB burden and indicators of TB programme efforts. To correct for spatial dependencies of the observations, the residuals of the multivariable models were tested for unexplained spatial autocorrelation. Spatial autocorrelation among the residuals was adjusted for by fitting a simultaneous autoregressive model (SAR).

**Results:**

Spatial clustering of TB CNRs was observed in all three countries. In Bangladesh, TB CNR were found significantly associated with testing rate (0.06%, *p* < 0.001), test positivity rate (14.44%, *p* < 0.001), proportion of bacteriologically confirmed cases (− 1.33%, *p* < 0.001) and population density (4.5*10–3%, *p* < 0.01). In Nepal, TB CNR were associated with population sex ratio (1.54%, *p* < 0.01), facility density (− 0.19%, *p* < 0.05) and treatment success rate (− 3.68%, *p* < 0.001). Finally, TB CNR in Pakistan were found significantly associated with testing rate (0.08%, *p* < 0.001), positivity rate (4.29, *p* < 0.001), proportion of bacteriologically confirmed cases (− 1.45, *p* < 0.001), vaccination coverage (1.17%, *p* < 0.001) and facility density (20.41%, *p* < 0.001).

**Conclusion:**

Subnational TB CNRs are more likely reflective of TB programme efforts and access to healthcare than TB burden. TB CNRs are better used for monitoring and evaluation of TB control efforts than the TB epidemic. Using spatial analytical techniques and profiling can help identify areas where TB is underreported. Applying these techniques routinely in the surveillance facilitates the use of TB CNRs in program planning.

## Background

Tuberculosis (TB) is an infectious respiratory disease which affects millions of people all over the world, but overwhelmingly affects the most vulnerable, hard to reach and socioeconomically disadvantaged people [1] . Despite efforts to drastically reduce the burden of TB, it remains within the top ten causes of deaths worldwide and outranks HIV/AIDS as one of the leading causes of death from an infectious disease [2]. In 2017, an estimated 10.0 million people fell ill from TB. However, only 6.4 million were notified to the national authorities. This means that an estimated 3.6 million people with TB were either not detected by the health system or not notified to the local authorities, and therefore missed by the formal health systems [2]. Many of these missing people with TB do not receive the care they need, leaving them vulnerable to develop severe and potentially fatal infections as well as being a potential source of transmission to those around them [1–3].

There are several barriers that TB patients may encounter which causes them to not be diagnosed or notified by the health systems. These barriers are well described by Uplekar et al. and others [4–11]. First of all, the patient needs to recognize the symptoms, but due to misperceptions, lack of knowledge or even social or internalized stigma, the patient might fail to do so [4–6]. Secondly, the patient needs to seek healthcare which can be compromised by distance to health facilities or transportation costs, loss of wages, costs for diagnosis and treatment and the perception of poor quality services [4, 7, 8]. Thirdly, health workers might fail to recognize the symptoms due to lack of training or lack of human resources [4, 9]. Fourth, the TB patient must be diagnosed as such, which can be complicated by insensitive screening and diagnostic tests, delay between testing and diagnosis that leads to loss to follow-up, or the inability for the individual with suspected TB to produce sputum [4, 10]. Fifth, the patient with TB might not initiate treatment due to direct and indirect treatment costs, geographical access to TB services, inadequate knowledge of the importance of timely treatment and stigma [11]{Citation}. Finally, due to poor knowledge of reporting procedures of the health care provider or poor engagement with the private health care sector, TB patients started on treatment might not be notified to the authorities [11].

TB case notification rates (CNR) are geographically heterogeneous [12–15]. Geographic variations in the presence of risk populations, TB transmission and burden are potential drivers of geographic variations in TB CNR. In Portugal for example, clusters of TB were associated with higher HIV/AIDS incidence, household crowding and incarceration [16]. Whereas in Brazil, higher rates of TB were associated with poor economic conditions, non-white population, urbanization and household crowding [17].

However, TB CNR are also a function of TB control efforts (i.e. the extent to which the health system effectively reaches out, diagnoses and notifies people with TB) and are therefore not necessarily an appropriate indicator of TB incidence. TB CNR can be only used as an indicator of TB incidence in places with a strong control program. A strong control program should take into account the subnational variation in TB burden and tailor its control efforts to the local epidemic [18].

It is our hypothesis that TB CNRs are only reflective of TB incidence in places where the TB control efforts are tailored to the local epidemic. Nonetheless, TB CNRs are often used as a proxy for TB incidence in the absence of a more reliable indicator of TB burden. If TB CNRs are used for the allocation of resources, we need a better understanding of what is driving the case notification rates. Therefore, the objective of this study is to gain better understanding of the drivers of subnational variations in TB notification rates in three South Asian countries with intermediate to high TB burden; Bangladesh, Nepal and Pakistan. The aim is to use spatial analytical techniques and profiling to gain better understanding of sociodemographic, health and programmatic indicators that underlie spatial variation of TB case notification rates.

## Methods

### Setting

This study uses aggregated subnational data from Bangladesh, Nepal and Pakistan. These countries were purposely selected based on the availability of recent subnational data on administrative areas (i.e. spatial data), TB notification, TB program efforts and indicators of demography and health; as well as their geographical proximity and thus similarities in geographic context. The study focusses on TB notification data from 2014 and 2015.

### Data

Data on TB case notification and programmatic indicators routinely collected by the National Tuberculosis Programs (NTPs) of Bangladesh (2015), Nepal (2014) and Pakistan (2015) were obtained for the most recent and complete year. Additional data on demography and health were derived from publicly available reports, such as the Demographic and Health Survey (DHS), Multiple Indicator and Cluster Survey (MICS), population census reports and statistical yearbooks [19–31] . These reports were collected online from governmental websites such as the USAID and the Bureau of Statistics of each country [32–35] . Only reports with subnational data were included and reports prior to 2010 were excluded from this study. One dataset was made per country, which included all available data on district level or higher, leading to three datasets: one dataset covering 64 administrative units of Bangladesh, one dataset covering 75 administrative units of Nepal and one dataset covering 142 administrative units of Pakistan.

To be able to map and visualise TB indicators, spatial data files of the respective administrative boundaries for each of the three countries were obtained from online spatial data repositories [36–38]. As subnational boundaries and administrative units change throughout the years, adjustments to the spatial data files were made (i.e. merging of 2 administrative units into 1) to ensure that the spatial data is the same as the TB reporting units.

### Variables

Subnational, annual TB case notification rates were used as the primary outcome variable for all analyses. It is defined as the number of reported cases per 100,000 population. It includes all reported cases, independent of the type of TB, the diagnostic method that had been used, or the patient’s TB history [39].

The covariates that were used for this study were selected foremost because of their known association to TB burden or TB programme performance, but also on the availability of these indicators in public reports. The covariates that were used for this study can be grouped into four broader themes. An overview of all covariates and their definitions can be found in Table 1.

1. Access to healthcare; the TB case notification rate tends to be higher in areas with better access to healthcare [40–42].
2. Socioeconomic status (SES); TB is associated with the socially and economically disadvantaged [17, 43].
3. Demography and key populations; Certain demographic and socio-economic key populations are known to have an increased risk of developing clinical TB, these include: miners, migrants, males, the malnourished and the elderly [44–48].
4. Quality of TB treatment and diagnostic services; The ability of the healthcare system to detect and treat TB patients is associated to various performance indicators [48, 49].

**Table 1:** Defintions of covariates

Theme	Covariate^**a**^	Definition
Access to healthcare		
Health facility density		Number of health facilities per 100,000 population.
Vaccination coverage		Percentage of fully immunized children between 12 and 23 months.
Under-five mortality rate		Number of deaths of children under five per 1000 live births.
Socioeconomic status		
Poverty headcount ratio		Percentage of the total population living below the national poverty line.
Literacy rate		Percentage of the population older than 15 who can read and write.
Demography & key populations		
Sex ratio		Number of males per 100 females.
Elderly population		Percentage of population over 65 years of age.
Ageing index		Number of persons aged 60 or over per 100 persons under the age of 15.
Stunting		Percentage of children under five with a height-for-age z-score below −2 standard deviations, from the median of the reference population.
Migrant distribution		Percentage of total internal migrant population per area.
Miners		Percentage of wage earners in mining industry/ total population in mining and quarrying industry.
Population density		Number of persons per square kilometre.
Security		Districts of Pakistan that were frequently reported as “insecure”.
Quality of TB diagnostic and treatment services		
Testing rate		Number of persons tested for TB per 100,000 population.
Test positivity rate		Percentage of tested individuals with a positive test result.
Bacteriologically diagnosed		Proportion of total cases with a bacteriologically confirmed test result.
Treatment success rate		Proportion of notified cases who have completed their treatment.

^a^Not all covariates were available for all countries

In order to link the covariate data to the units in the spatial data files, every geographical unit was assigned an unique identifier in the form of a numerical code. The same code was given to the spatial information belonging to that geographical unit. This way, the compiled subnational data were merged with the spatial information and subsequently visualized in GIS software.

### Statistical analysis

#### Spatial autocorrelation

The global univariable Moran’s I was used to test for the presence of spatial clustering (spatial autocorrelation) in the TB case notification rate at subnational level. In addition, Local Indicators of Spatial Autocorrelation (LISA) were calculated in order to identify and locate clusters of districts with a relatively high or low TB case notification rate. A first order queen contiguity connectivity matrix was used to define neighbouring districts. These statistics give valuable information about the independency (i.e. absence of spatial dependency) of observations, an important assumption in regression analyses [50].

#### Univariable and multivariable analyses

Generalized linear models (GLM) with log transformed outcome variable (TB CNR) were fitted to the data for the univariable and multivariable analyses - separately for each country - based on the fit and nature of the data, as disease rates often follow a log-normal distribution [51]. A poisson and negative binomial model were also considered as these are frequently used to model disease rates, but the lognormal model provided a better fit for all three countries. First, univariable analyses were conducted for each covariate to assess the strength and direction of the association. Next, all variables were included in a multivariable GLM Finally, simultaneous autoregressive (SAR) models were fitted to the data to account for unexplained spatial autocorrelation in the residuals of the multivariable GLM. This model uses a spatially correlated error structure based on a contiguity weights matrix taking into account only directly adjacent spatial neighbours (i.e. shared borders). The reported coefficients are exponentiated and reflect percentage change in TB CNR. All models are reported with corresponding 95% confidence interval (CI) and p- values, as well as the global Moran’s I statistic with corresponding *p*-value for the residuals of the multivariable models.

Pearson’s correlation coefficients were calculated for all covariates before multivariable modelling. If two variables were correlated (i.e. correlation coefficient exceeding 0.7), one of the variables was excluded from further analysis. The variable to be excluded was determined by the number of correlations, where the variable with highest number of correlations was excluded. In case both variables had equal numbers of correlations than the variable with a non-significant result in the univariable analysis was excluded.

Data analyses were performed using GeoDa version 1.8.16.4 for the assessment of spatial autocorrelation, RStudio version 1.0.143 for all other statistical analyses and QGIS version 2.18.4 for the geographical visualization of the data [52–54].

## Results

### Descriptive analysis

A total of 205,98; 37,025 and 326,152 people were diagnosed with TB and notified in Bangladesh (2015), Nepal (2014) and Pakistan (2015) respectively. Subnational TB case notifications per hundred thousand population ranged from 50 to 187 in Bangladesh, from 31 to 227 in Nepal and from 9 to 689 in Pakistan (Table 2, 3 and 4).

**Table 2:** Descriptive statistics for Bangladeshi districts

Variable	N	Mean^**a**^	SD	Min	Max	Missing		National^**b**^
						N	%	
Population	64	2,488,828	2,039,092	449,100	14,386,878	0	0.0	159,284,969
Case notification	64	3219	3291	455	23,686	0	0.0	205,985
Case notification rate	64	124	33	50	187	0	0.0	129
Healthcare access								
Facility density (per 100,000 population)	64	0.9	0.9	0.3	5.6	0	0.0	0.7
Under-five mortality rate (per 1000 live births)	64^c^	46	9	35	67	0	0.0	N/A
Vaccination coverage (%)	64	87.2	3.8	76.4	94.5	0	0.0	N/A
Socioeconomic status								
Poverty headcount ratio (%)	64	32.3	12.1	3.6	63.7	0	0.0	N/A
Literacy rate (%)	64	54.7	7.8	37.5	73.7	0	0.0	N/A
TB programme efforts								
Bacteriologically confirmed (%)	64	74.1	7.3	56.9	89.1	0	0.0	72.5
Test positivity rate (%)	64	6.3	1.4	3.3	10.7	0	0.0	6.5
Test rate (per 100,000 population)	64	1199	352	569	2015	0	0.0	1145
Treatment success rate (%)	64	94.4	2.3	87.1	99.2	0	0.0	94.5
Demography								
Stunting (%)	64	41.2	7.0	27.7	55.9	0	0.0	N/A
Sex ratio (male: female)	64	99.2	4.8	90.2	119.3	0	0.0	N/A
Population density (per km^2^)	64	1108	1038	86	8111	0	0.0	
Elderly population (%)	64	7.8	1.3	4.8	11.3	0	0.0	N/A
Migrant population (%)^c^	64	0.06	0.06	0.01	0.39	0	0.0	N/A

^a^Unweighted mean of districts. ^b^Weighted national average. ^c^Data were available on the first administrative level only

*N/A* National average could not be calculated due to lack of denominator

**Table 3:** Descriptive statistics for Nepali districts

Variable	N	Mean^**a**^	SD	Min	Max	Missing		National^**b**^
						N	%	
Population	75	364,160	304,925	5284	1,931,225	0	0.0	27,311,978
Case notification	75	494	572	4	3642	0	0.0	37,025
Case notification rate	75	110	49	31	227	0	0.0	136
Healthcare access								
Facility density (per 100,000 population)	75	98.9	73.6	24.7	530.0	0	0.0	65.6
Under-five mortality rate (per 1000 live births)	75^c^	64	9	55	82	0	0.0	N/A
Vaccination coverage (%)	75^c^	88.9	4.7	79.8	97.4	0	0.0	N/A
Socio-economic status								
Poverty headcount ratio (%)	75	27.7	13.4	4.0	64.1	0	0.0	N/A
Literacy rate (%)	75	58.1	10.6	34.6	87.3	0	0.0	N/A
Demography								
Stunting (%)	75^c^	43.4	9.7	31.3	59.5	0	0.0	N/A
Sex ratio (male: female)	75	92.7	9.0	76.0	127.3	0	0.0	N/A
Population density (per km^2^)	75	312	590	3	4416	0	0.0	N/A
Ageing index (elders per 100 persons under 15)^d^	74	16.3	6.1	6.2	35.5	1	1.3	N/A
Miners	75^c^	1	0.5	0.3	1.7	0	0.0	N/A
Migrant distribution (%)	75^c^	35.9	5.0	26.0	39.3	0	0.0	N/A
TB programme efforts								
Bacteriologically confirmed (%)	75	72.2	12.8	22.2	100.0	0	0.0	70.3
Treatment success rate (%)	74	92.4	4.8	81.7	100.0	1	1.3	90.7

^a^Unweighted mean of districts. ^b^Weighted national average. ^c^Data were available on the regional level only

N/A: National average could not be calculated due to lack of denominator. ^d^ The number of elders per 100 persons younger than 15

**Table 4:** Descriptive statistics for Pakistani districts

Variable	N	Mean^**a**^	SD	Min	Max	Missing		National^**b**^
						N	%	
Population	142	1,310,675	1,686,617	26,265	14,040,575	0	0.0	186,115,787
Case notification	142	2297	3286	21	22,185	0	0.0	326,153
Case notification rate	142	147	93	9	689	0	0.0	175
Healthcare access								
Facility density (per 100,000 population)	132	1.3	1.0	0.4	8.9	10	7.0	0.8
Under-five mortality rate (per 1000 live births)	122^c^	95.6	15.7	43.0	111.0	20	14.1	N/A
Vaccination coverage (%)	122	69.9	23.0	3.0	99.0	20	14.1	N/A
Socio-economic status								
Poverty headcount ratio (%)	114	45.3	24.2	3.7	96.4	28	19.7	N/A
Literacy rate (%)	122	49.9	14.5	19.6	85.0	20	14.1	N/A
Demography								
Stunting (%)	92^c^	44.1	7.7	22.2	56.7	50	35.2	N/A
Migrant distribution (%)	115^c^	28.5	28.0	0.7	67.8	27	19.0	N/A
TB programme efforts								
Bacteriologically confirmed (%)	141	54.0	15.3	6.3	100.0	1	0.7	52.0
Test positivity rate (%)	139	14.7	6.6	1.9	41.7	3	2.1	13.8
Test rate (per 100,000 population)	139	478	498	40	4369	3	2.1	542
Treatment success rate (%)	141	92.6	5.7	75.0	100.0	1	0.7	91.8

^a^Unweighted mean of districts. ^b^Weighted national average. ^c^Data were available on the first administrative level only

*N/A* National average could not be calculated due to lack of denominator

**Fig. 1:**
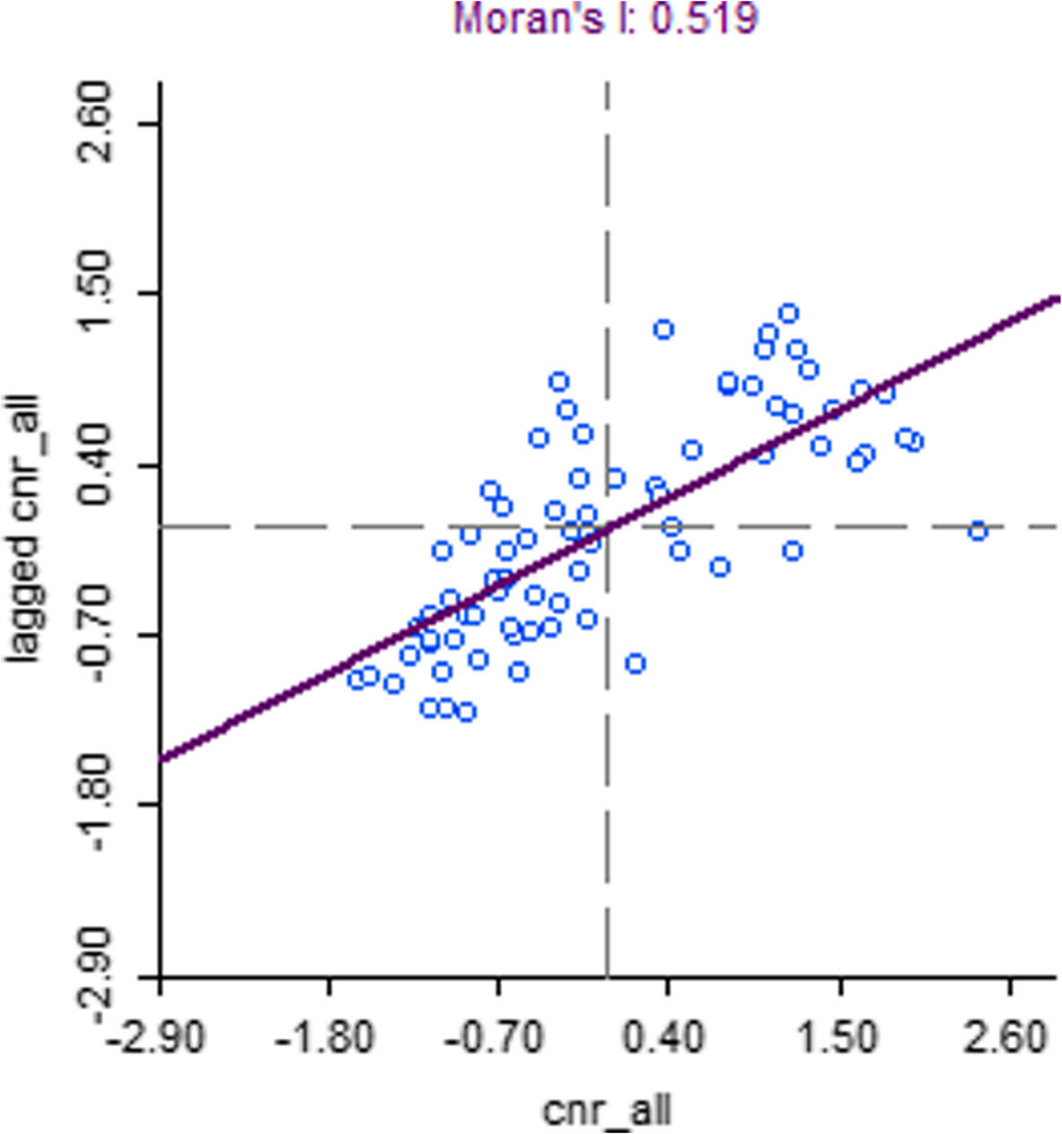
Moran’s I plot showing the strength of spatial autocorrelation of TB CNR (2014), Nepal

**Fig. 2:**
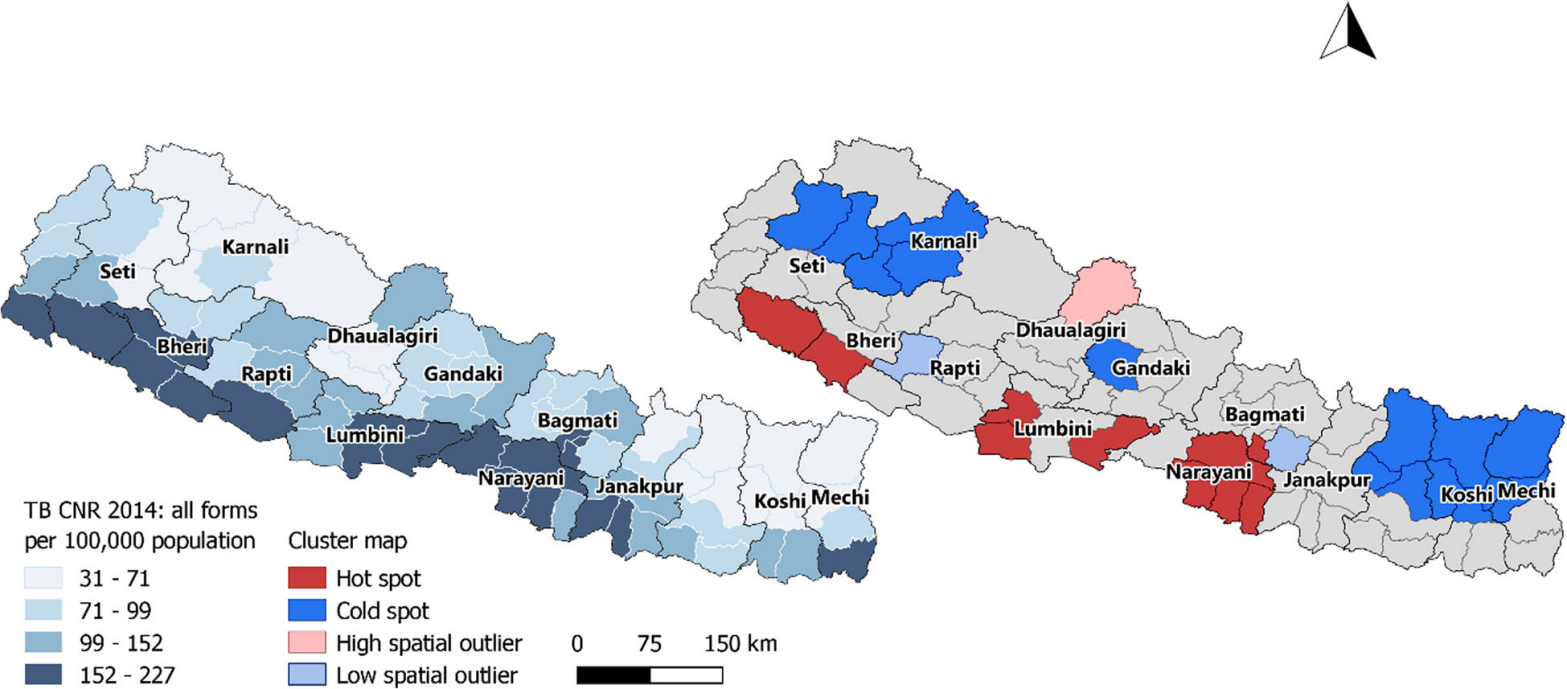
Map of Nepal showing the geographical variations and spatial clustering of the TB CNR 2014

**Fig. 3:**
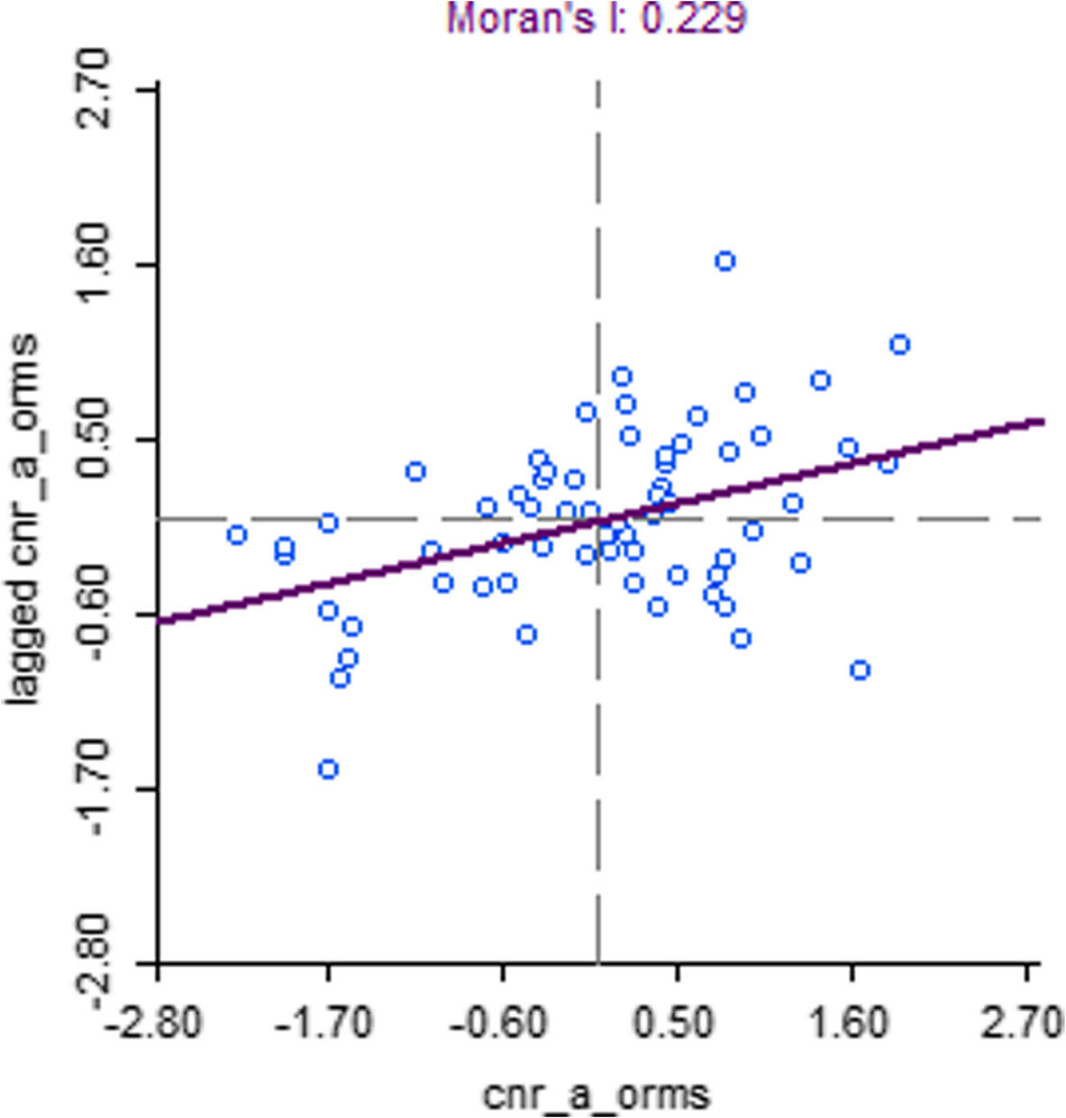
Moran’s I plot showing the strength of spatial autocorrelation of TB CNR (2015), Bangladesh

**Fig. 4:**
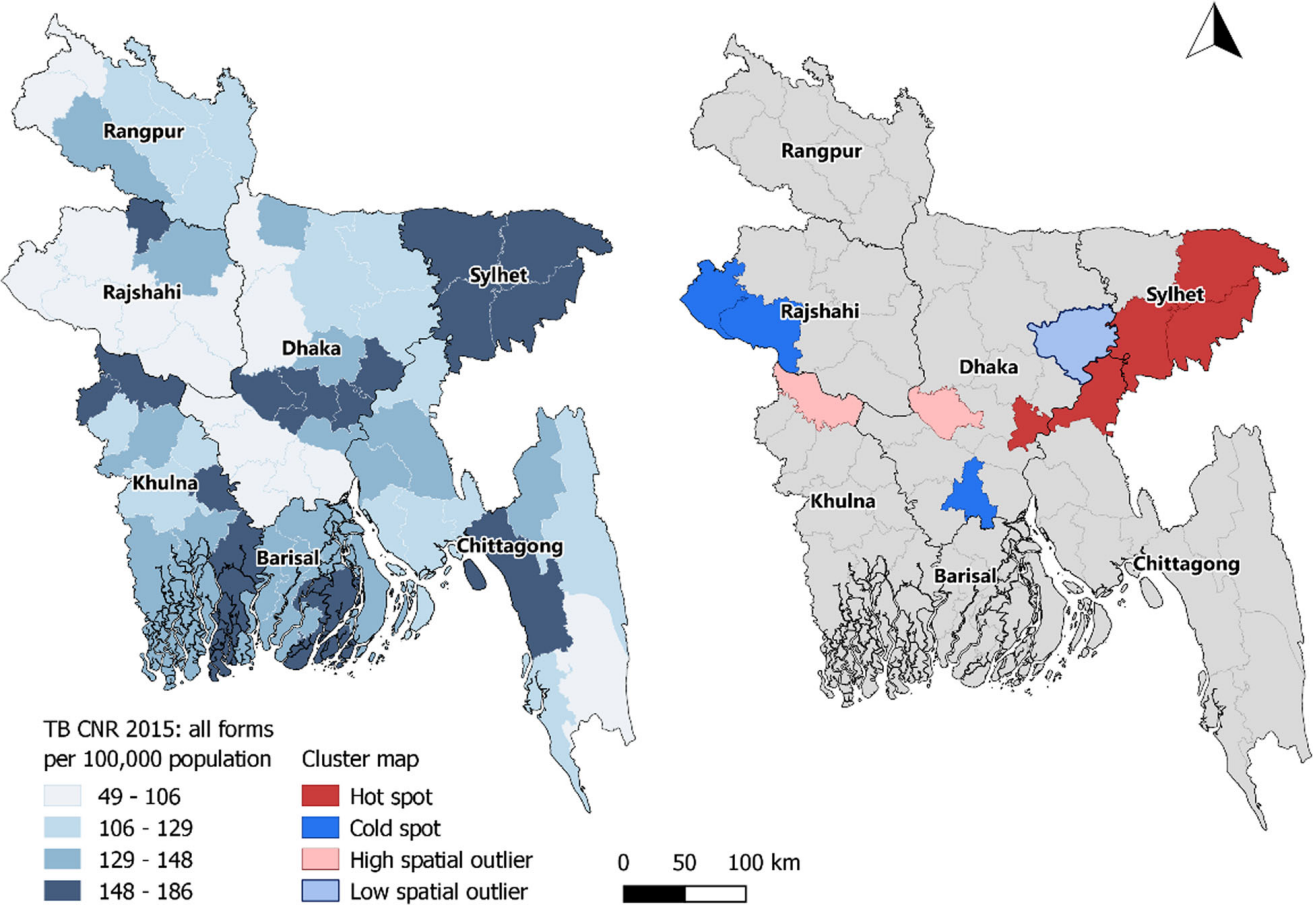
Map of Bangladesh showing the geographical variations and spatial clustering of the TB CNR 2015

Spatial clustering of TB case notification rates was observed in Nepal (Moran’s I: 0.52, *p*-value < 0.001, Fig. 1). In the northern mountainous region of Nepal clusters of low notification rates (cold spots) were observed whereas in the lower “Terai” region in the south of Nepal several clusters of high notification rates (hot spots) were identified (Fig. 2) Moderate spatial clustering of TB case notification rates were observed in Bangladesh (Moran’s I: 0.23, *p*-value < 0.01, Fig. 3), with one larger hot spot in the eastern division Sylhet and two small cold spots in Rajshahi and Dhaka divisions (Fig. 4).

**Fig. 5:**
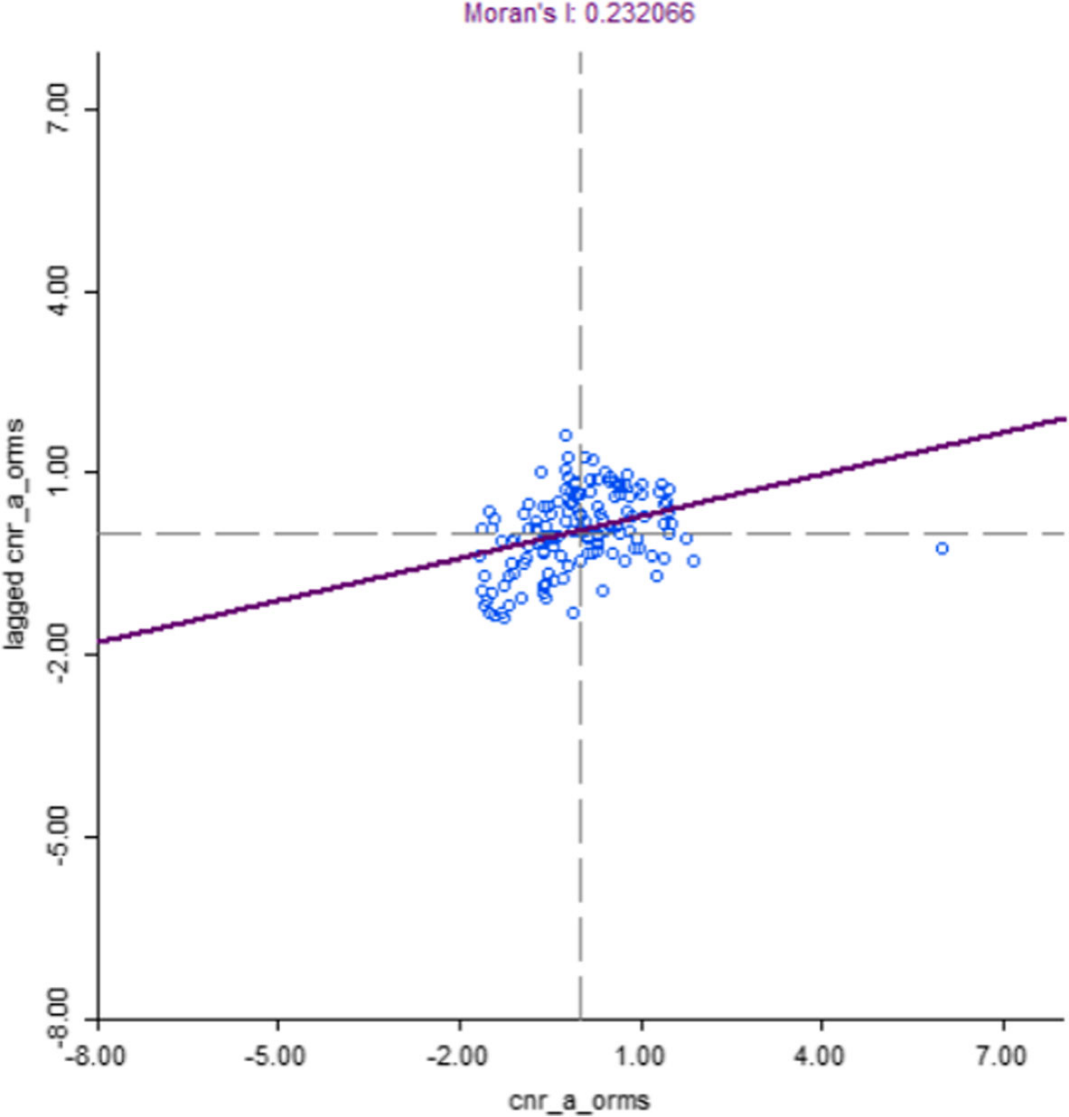
Moran’s I plot showing the strength of spatial autocorrelation of TB CNR (2015), Pakistan

**Fig. 6:**
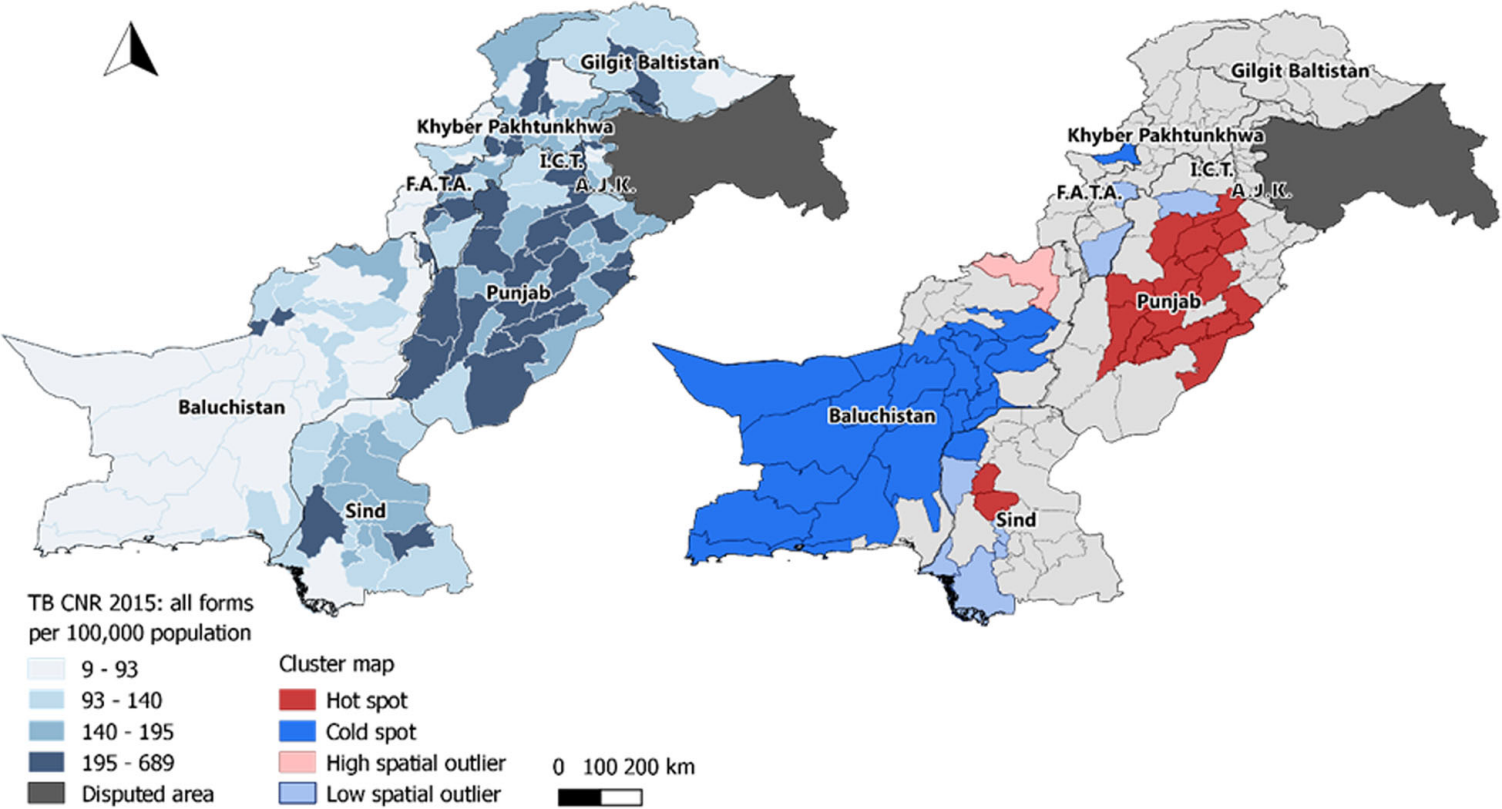
Map of Pakistan showing the geographical variations and spatial clustering of the TB CNR 2015

Moderate spatial clustering was also observed for Pakistan (Moran’s I: 0.23, *p*-value < 0.001, Fig. 5), with a large cold spot covering almost the entire province of Balochistan and one major hot spot in Punjab province (Fig. 6).

### Statistical results

Tables 5, 6 and 7 show the results of the univariable, non-spatial multivariable and spatial multivariable models of the natural logarithm of the TB case notification rate for Bangladesh, Nepal and Pakistan.

**Table 5:** Results of the univariable and multivariable analyses for Bangladesh and global Moran’s I of the residuals of the multivariable model

Covariate	Univariable		Non-spatial multivariable ^**a**^	
	B	CI	B	CI
Constant	–	–	52.12	9.74–261.59
Population density	0.01	− 0.002 – 0.01	4.5e-3**	1.7e-3 – 7.3e-3
Bacteriologically diagnosed	−1.51**	−2.45 – − 0.57	−1.33***	− 1.65 - -1.01
Testing rate	0.05***	0.03–0.07	0.06***	0.06–0.07
Positivity rate	12.83***	7.97–8.52	14.44***	12.44–16.48
Facility density	0.37	−7.17 – 8.51	–	–
Treatment success rate	5.87***	2.82–8.99	− 0.25	− 1.52 - 1.04
Vaccination coverage	− 1.22	−3.12 – 0.72	0.34	−0.39 - 1.08
Literacy rate	0.23	−0.07 – 1.20	−0.2	− 0.54 - 0.13
Poverty headcount ratio	−0.62*	−1.21 – − 0.2	−0.05	− 0.24 - 0.15
Stunting	0.56	−0.50 – 1.63	−0.02	− 0.36 - 0.32
Under-five mortality ratio	1.15**	0.31–1.99	− 0.05	−0.38 - 0.27
Sex ratio	0.53	−1.01 – 2.09	0.14	−0.58 - 0.86
Elderly population	−1.83	−7.17 – 3.82	1.11	−1.58 - 3.88
Migrant distribution	0.88	−0.32 – 2.10	–	–
Global Moran’s I of the residuals			−0.07	
*P*-value of global Moran’s I			0.28	

^a^Generalized linear model for the natural logarithm of the TB case notification rate; not adjusted for spatial autocorrelation

All coefficients (B) are exponentiated and reflect percentage change in TB case notification rate

* *p* < 0.05, ** *p* < 0.01, *** *p* < 0.001

**Table 6:** Results of the univariable and multivariable analyses for Nepal and global Moran’s I of the residuals of the multivariable models

	Univariable		Non-spatial multivariable ^**a**^		Spatial multivariable ^**b**^	
Covariate	B	CI	B	CI	B	CI
Constant	–	–				
Facility density	− 0.29***	− 0.42 – − 0.16	− 0.19	− 0.39 - 0	− 0.19*	− 0.37 - -0.01
Sex ratio	0.99	−0.18 – 2.18	1.56*	0.31–2.83	1.54**	0.40–2.69
Treatment success rate	−5.19***	−6.95 – −3.40	− 3.66***	−5.38 - -1.91	−3.68***	−5.27 - -2.08
Stunting	−1.83***	−2.83 – − 0.83	− 1.89*	− 3.27 - -0.48	− 1.88**	−3.15 - -0.60
Bacteriologically diagnosed	− 0.63	−1.45 – 0.18	− 0.32	− 1.06 - 0.43	−0.31	− 0.99 - 0.37
Population density	0.03***	0.01–0.05	0	−0.02 - 0.01	0	−0.02 - 0.01
Migrant distribution	−0.43	− 2.52 – 1.71	−1.31	−3.59 - 1.02	− 1.36	− 3.47 - 0.8
Literacy rate	0.04	−0.97 – 1.05	1.21	−0.16 - 2.6	1.16	−0.11 - 2.44
Under-five mortality rate	0.45	−0.68 – 1.60				
Poverty headcount ratio	−0.89*	−1.57 – − 0.04	57.39	−41.65 - 324.53	50.99	−39.55 - 277.15
Ageing index	−1.07	−2.79 – 0.67	−0.58	−3.28 - 2.19	−0.56	−3.06 - 2.01
Miners	−20.88*	−35.76 – − 2.54				
Vaccination coverage	−1.29	−3.48 – 0.95	− 1.2	− 3.45 - 1.1	−1.23	−3.29 - 0.88
Global Moran’s I of the residuals			0.17		−0.02	
*P*-value of global Moran’s I			0.01		0.44	

^a^Generalized linear multivariable model for the natural logarithm of the TB case notification rate; not adjusted for spatial autocorrelation

^b^Simultaneous autoregressive multivariable model for the natural logarithm of the TB case notification rate; adjusted for spatial autocorrelation

All coefficients (B) are exponentiated and reflect percentage change in TB case notification rate

* *p* < 0.05, ** *p* < 0.01, *** *p* < 0.001

**Table 7:** Results of the univariable and multivariable analyses for Pakistan and global Moran’s I of the residuals of the multivariable models

	Univariable		Non-spatial multivariable ^**a**^		Spatial multivariable ^**b**^	
Covariate	B	CI	B	CI	B	CI
Constant	–	–	25.00***	25.60–60.60	24.06***	23.60–57.97
Testing rate	0.04***	0.02–0.07	0.08***	0.05–0.11	0.08***	0.05–0.10
Test positivity rate	4.04***	2.20–5.91	4.32***	2.6–6.08	4.29***	2.73–5.87
Bacteriologically diagnosed	−1.84***	−2.58 – − 1.10	−1.43***	− 2.06 - -0.8	−1.45***	−2.02 - -0.88
Treatment success rate	0.99	0.97–1.02	0.06	−1.45 - 1.59	0.07	−1.34 - 1.5
Vaccination coverage	1.88***	1.38–2.38	1.15***	0.52–1.78	1.17***	0.61–1.72
Migrant distribution	1.5***	1.06–1.9	0.61	−0.63 - 1.86	0.64	−0.51 - 1.81
Facility density	−9.19	−20.01 – 3.10	20.64**	7.94–34.84	20.41***	8.42–33.72
Poverty headcount ratio	−1.87***	− 90.40 – −75.37	6.42	−36.05 - 77.09	16.56	−25.55 - 82.48
Stunting	−1.27*	−2.47 – −0.05	1.55	−1.18 - 4.37	1.52	−0.94 - 4.04
Under-five mortality rate	−1.86**	−2.15 – −0.47	−0.55	− 2.19 - 1.1	− 0.55	−2.06 - 0.99
Security	− 0.11	− 0.43 – − 0.21	−7.35	−45.33 - 56.99	− 7.85	−42.66 - 48.11
Global Moran’s I of the residuals			0.11		−0.04	
*P*-value of global Moran’s I			0.01		0.14	

^a^Generalized linear multivariable model for the natural logarithm of the TB case notification rate; not adjusted for spatial autocorrelation

^b^Simultaneous autoregressive multivariable model for the natural logarithm of the TB case notification rate; adjusted for spatial autocorrelation

All coefficients (B) are exponentiated and reflect percentage change in TB case notification rate

* *p* < 0.05, ** *p* < 0.01, *** *p* < 0.001

Based on the correlation matrices (Additional file 1) and the univariable analyses, the following covariates were excluded prior to multivariable modelling: facility density and migrant population (Bangladesh), miners and under 5 mortality rate (Nepal), and literacy rate (Pakistan).

#### Bangladesh

The spatial GLM model for Bangladesh showed that TB CNR is positively associated to population density (β = 4.5e-3; 95%CI: 1.7e-3 - 7.3e-3), testing rate (β = 0.06, CI: 0.06–0.07) and positivity rate (β = 14.44, CI: 12.44–16.48). Furthermore, the model suggests that an increase in the proportion of bacteriologically confirmed cases is associated with a decrease in TB case notification rate of 1.33% (CI: − 1.65 - -1.01). The model fully accounted for spatial autocorrelation in TB CNR (Moran’s I: − 0.07, *p*-value = 0.28), therefore no SAR model was fitted.

#### Nepal

The residuals of the non-spatial multivariable model for Nepal were found to spatially auto correlate (Moran’s I: 0.17, *p*-value < 0.01) and a spatial model correcting for the special dependencies was computed. The spatial multivariable model shows that facility density is inversely associated to the TB CNR, where an increase of one unit in facility density is associated with a decrease of the TB CNR of 0.19% (CI: − 0.37 - -0.01). Furthermore, an increase of one unit in sex ratio is associated with an increase in TB CNR of 1.54% (CI: 0.40–2.69). Treatment success rate (β:-3.68, CI:-5.27 - -2.08) and stunting (β:-1.88, CI: − 3.15 - -0.60), are both inversely associated to TB CNR in the spatial model. The spatial model accounted for all spatial autocorrelation (Moran’s I: − 0.02, *p*-value = 0.44).

#### Pakistan

According to the spatial multivariable model TB CNR (which was necessary with a Moran’s I: 0.11 after the GLM) was found positively associated with testing rate (β = 0.08, CI: 0.05–0.10), test positivity rate (β = 4.29, CI: 2.73–5.87), proportion bacteriologically diagnosed (β = − 1.45, CI: − 2.02 – − 0.88), vaccination coverage (β = 1.17, CI: 0.61–1.72) and facility density (β = 20.41, CI: 8.42–33.72). The SAR model fully accounted for the spatial autocorrelation of the TB CNR (Moran’s I: − 0.04, *p*-value = 0.14).

## Discussion

From TB prevalence studies we know that there is a gap between TB notification and TB burden. Onozaki et al. assessed national TB prevalence surveys in Asia from 1990 to 2012 and found the TB prevalence to be twice as high as the number of notified cases [55]. Yet TB notification rates are often used as a measure of TB incidence, making it imperative that we have a better understanding of the drivers of TB notification rates.

The data and results presented in this paper show that TB case notifications across the three countries analysed are spatially heterogeneous and spatially clustered. The positive association between population density in Bangladesh and sex ratio in Nepal suggest that part of the variation in TB CNR can be explained by proxies for TB risk. In Bangladesh an increase in population density is positively associated with TB CNR (B:4.5e-3, 95%CI: 1.7e-3 – 7.3e-3), a higher population density is indicative of more crowding which increases transmission of TB [56]. Crowding has been associated with increased risk of TB in Bangladesh in both adults and children [57, 58]. Furthermore, a positive association between sex ratio and TB CNR in Nepal (B:1.54, 95%CI:0.40–2.69) shows that more TB patients are diagnosed in districts with more men. This is line with findings of the TB prevalence survey, which found a much higher prevalence in men [59].

However, the models also suggest that part of the variation in TB CNR can be explained by programmatic factors. TB CNR is inversely associated with the proportion of TB patients with a bacteriologically confirmation in both Bangladesh (B:-1.33, 95%CI:-.65 - -1.01) and Pakistan (B:-1.45, 95%CI: − 2.02 - -0.88). One explanation is that more sensitive diagnostic methods can result in a reduction of clinically diagnosed pulmonary TB, which may cause a reduction in the overall TB CNR [60].

The positive association between testing rate and TB CNR in Bangladesh (B:0.06, 95%CI:0.06–0.07) and Pakistan (B:0.08, 95%CI:0.05–0.10) suggests that more testing yields higher notification. In a well-adjusted system, the level of testing is direct response to the local TB burden. But the positive association between test positivity rates in both countries (Bangladesh: B:14.44, 95%CI:12.44–16.48, Pakistan B:4.29, 95%CI:2.73–5.87) suggests otherwise. When the testing rate increases, one would expect the positivity rate to decrease or remain stable. The increasing test positivity rate suggests that the current level of testing does not meet the local need for testing or that testing efforts may be targeted towards populations more likely to suffer from TB.

The positive association between TB CNR with facility density (B:20.41, 95%CI:8.42–33.72) and vaccination coverage (B:1.17, 95%CI:0.61–1.72) in Pakistan substantiate the results above. Facility density and vaccination coverage are both indicative of better access to health care and improved uptake of health services [61].

In contrast with the findings above, facility density is inversely associated with TB CNR in Nepal (B:-0.19, 95%CI:-0.37 - -0.01). One explanation is that in areas with very low population numbers (such as the hilly and mountainous regions in Nepal), the facility density is likely to be very high. Likewise, in highly urbanized areas (e.g. Kathmandu) the facility to population ratio can be very low due to the high population denominator. Although the placement of health facilities takes into account population density and the unmet need of the population, the facility to population ratio calculated on district level does not reflect this and might therefore not be the right metric to assess access to healthcare on a district level.

The inverse association between treatment success rate and TB CNR in Nepal (B:-3.68, 95%CI:-5.27 - -2.08) suggests that higher notification rates negatively affect case management, possibly due a higher burden on the health system to follow-up on TB patients or to provide the required medication.

The TB CNR decreases with 1.88% (95%CI: − 3.15 - -0.60) with increasing prevalence of stunting (Nepal). Stunting is strongly associated with lower socioeconomic status and poor health, which increases the risk of TB, but in this case may reflect lower access to healthcare. This is in line with findings from the TB prevalence survey, where the proportion of TB patients not seeking care was higher among the poor [59].

Although the models largely agree with one another, we see differences in the associations between sociodemographic and access indicators with the CNR. This finding underscore that the results are to some extent influenced by country context, specifically health care infrastructure and TB epidemiology.

### Strengths and limitations

We were able to combine data from multiple sources, hence capturing different dimensions of the same phenomenon as well as minimizing inadequacies that occur in data from a single source. Furthermore, the models are congruent in what they suggest and the strength and direction of the associations are also consistent between the models. These similarities between independent models decrease the likelihood of the observed associations being the result of chance. In addition, most of the spatial clustering is adjusted for in the final models.

The data that were used in this study were derived from various sources and were therefore available on different administrative levels and for different years. However, we expect demographic indicators such as poverty, migration and population size to remain stable over the course of three to 5 years or to grow proportionally over time. Although natural disasters and conflict are known to influence demographic indicators, these changes are often not reflected in district level population statistics as presented by national statistical offices which also cover a broader time frame. However, the possibility remains that data from different years does not accurately reflect the situation in the year from which TB data were available. Furthermore, some data were only available on a higher administrative level (e.g. province or regional). These data could not be used to reflect the district-level variations that this study is trying to address. In addition, these data points are not independent from one another, which increases the risk of a type II error. Finally, subnational data on HIV or TB prevalence were not available for this study and could therefore not be included.

## Conclusion

The results give clear indications of spatial clustering of the tuberculosis case notification rates in Bangladesh, Nepal and Pakistan. Where this is a result that is often found by other research concerning spatial epidemiology of TB, most of these studies attribute this to social and demographic indicators and neglect the influence that TB program efforts might have.

The result of this study show that notification of TB is mainly associated with access to healthcare and TB program efforts. This is not necessarily a problem in case the local efforts are a direct response to the TB burden, in which TB notification rates can be used as a proxy for TB incidence. However, the associations that were found do not suggest that the TB program efforts are a response to TB burden. In fact, they suggest that TB notification is dependent on programmatic response such as the ability to test, diagnose and treat individuals, but also the ability of patients to access health care. Hence, TB notifications should not be used as a proxy for TB incidence.

However, TB notifications are a great source of information if they are interpreted in the context of the local health system. As such, assessing changes over time in the geographical distribution of TB notification rates can be useful to monitor changes in policy, interventions or programmatic efforts. Spatial analytical techniques and profiling allows for the identification of spatial outliers and local inconsistencies which can be indicative of TB under notification. This valuable information can be used to prioritize areas which require further supervision and tailor interventions according to their local needs in an effort to diagnose and successfully treat the missing people with TB.

## Supplementary information

**Additional file 1.** Correlation Matrices of Bangladesh, Nepal and Pakistan. Three individual correlation matrices for Bangladesh, Nepal and Pakistan.

## Data Availability

Part of the data used for this study comes from publicly available documents such as the Demographic and Health Survey or reports from National Statistical Offices. All publicly available documents used for this study can be found in the references of this article. All TB data that support the finding of this study are available from the National TB programs from which they were retrieved but restrictions apply to the availability of these data, which were used under license for the current study, and so are not publicly available. Data are however available from the authors upon reasonable request and with permission of the National TB programs.

## Abbreviations

CI: Confidence interval
CNR: Case notification rate
DHS: Demographic and Health Survey
Gi: Global Moran’s I
GIS: Geographical Information System
GLM: Generalized Linear Model
HIV: Human Immunodeficiency Virus
LISA: Local Indicators of Spatial Autocorrelation
MICS: Multiple Indicator Cluster Survey
NTP: National TB Program
SAR: Simultaneous Autoregressive model
SES: Socio economic status
TB: Tuberculosis
