# Supplementary material for: Finding gaps in TB notifications: spatial analysis of geographical patterns of TB notifications, associations with TB program efforts and social determinants of TB risk in Bangladesh, Nepal and Pakistan
Source: BMC Infect Dis. 2020 Jul 10;20:490. doi: 10.1186/s12879-020-05207-z (PMC7350590; doi:10.1186/s12879-020-05207-z)
Supplement: Supplementary file 1 — Additional file 1. Correlation Matrices of Bangladesh, Nepal and Pakistan. Three individual correlation matrices for Bangladesh, Nepal and Pakistan. [file 12879_2020_5207_MOESM1_ESM.docx]

# Appendix A. Pearson’s correlation matrices for Bangladesh, Nepal and Pakistan

*Correlation matrix 1. Bangladesh*

|  | **1.** | **2.** | **3.** | **4.** | **5.** | **6.** | **7.** | **8.** | **9.** | **10.** | **11.** | **12.** | **13.** | **14.** |
| --- | --- | --- | --- | --- | --- | --- | --- | --- | --- | --- | --- | --- | --- | --- |
| 1. Bacteriologically diagnosed | - |  |  |  |  |  |  |  |  |  |  |  |  |  |
| 2. Testing rate | 0.07 | - |  |  |  |  |  |  |  |  |  |  |  |  |
| 3. Migrant population | -0.04 | 0.03 | - |  |  |  |  |  |  |  |  |  |  |  |
| 4. Population density | -0.21 | -0.18 | 0.72*** | - |  |  |  |  |  |  |  |  |  |  |
| 5. Stunting | -0.23 | -0.07 | -0.09 | -0.01 | - |  |  |  |  |  |  |  |  |  |
| 6. Vaccination | 0.16 | 0.01 | -0.20 | -0.19 | -0.27* | - |  |  |  |  |  |  |  |  |
| 7. Under-five mortality rate | -0.14 | 0.19 | -0.01 | -0.13 | 0.01 | -0.38** | - |  |  |  |  |  |  |  |
| 8. Poverty headcount ratio | 0.14 | -0.09 | -0.29* | -0.19 | 0.06 | 0.17 | -0.26* | - |  |  |  |  |  |  |
| 9. Sex ratio | 0.07 | 0.09 | 0.74*** | 0.43*** | 0.00 | -0.33** | 0.01 | -0.26* | - |  |  |  |  |  |
| 10. Elderly population | 0.04 | 0.06 | -0.38** | -0.28* | -0.19 | 0.45*** | -0.14 | 0.19 | -0.68*** | - |  |  |  |  |
| 11. Treatment success rate | -0.04 | 0.47*** | 0.13 | -0.06 | 0.10 | -0.11 | 0.41*** | -0.20 | 0.15 | -0.30* | - |  |  |  |
| 12. Test positivity rate | -0.12 | -0.15 | 0.07 | 0.20 | 0.20 | -0.21 | 0.29* | -0.18 | 0.00 | -0.18 | 0.20 | - |  |  |
| 13. Literacy rate | 0.02 | 0.12 | 0.42*** | 0.30* | -0.22 | 0.27* | -0.12 | -0.13 | 0.04 | 0.22 | 0.07 | -0.07 | - |  |
| 14. Facility density | -0.28* | -0.72*** | -0.01 | 0.25* | 0.07 | -0.04 | -0.03 | -0.09 | -0.18 | 0.02 | -0.30* | 0.24 | -0.11 | - |

* p<0.05, ** p<0.01, ***p<0.001.

*Correlation matrix 2. Nepal*

|  | 1. | 2. | 3. | 4. | 5. | 6. | 7. | 8. | 9. | 10. | 11. | 12. | 13. |
| --- | --- | --- | --- | --- | --- | --- | --- | --- | --- | --- | --- | --- | --- |
| 1. Bacteriologically diagnosed | - |  |  |  |  |  |  |  |  |  |  |  |  |
| 2. Stunting | -0.08 | - |  |  |  |  |  |  |  |  |  |  |  |
| 3. Vaccination coverage | 0.32*** | -0.18** | - |  |  |  |  |  |  |  |  |  |  |
| 4. Under-five mortality | -0.12 | 0.59*** | -0.02 | - |  |  |  |  |  |  |  |  |  |
| 5. Poverty headcount ratio | -0.11 | 0.70*** | -0.27*** | 0.69*** | - |  |  |  |  |  |  |  |  |
| 6. Miners | 0.28*** | -0.46*** | 0.12 | -0.83*** | -0.52*** | - |  |  |  |  |  |  |  |
| 7. Sex ratio | -0.21** | 0.21** | -0.55*** | 0.04 | 0.20** | -0.19** | - |  |  |  |  |  |  |
| 8. Ageing index | 0.34*** | -0.37*** | 0.20** | -0.60*** | -0.49*** | 0.56*** | -0.13* | - |  |  |  |  |  |
| 9. Migrant distribution | 0.30*** | -0.50*** | -0.03 | -0.54*** | -0.36*** | 0.73*** | -0.08 | 0.55*** | - |  |  |  |  |
| 10. Treatment success rate | 0.25*** | -0.09 | 0.01 | -0.05 | 0.02 | 0.08 | -0.11 | -0.11 | 0.09 | - |  |  |  |
| 11. Literacy rate | 0.25*** | -0.35*** | 0.35*** | -0.30*** | -0.56*** | 0.32*** | -0.13 | 0.47*** | 0.21** | -0.12 | - |  |  |
| 12. Facility density | -0.10 | -0.53*** | -0.05 | 0.00 | -0.37*** | -0.01 | 0.14* | -0.17* | 0.10 | -0.05 | 0.24*** | - |  |
| 13. Population density | -0.05 | -0.37*** | -0.09 | -0.16* | -0.35*** | 0.03 | 0.32*** | 0.03 | 0.19** | -0.08 | 0.36*** | 0.45*** | - |
| * p<0.05, ** p<0.01, ***p<0.001. |  |  |  |  |  |  |  |  |  |  |  |  |  |

*Correlation matrix 3. Pakistan*

|  | **1.** | **2.** | **3.** | **4.** | **5.** | **6.** | **7.** | **8.** | **9.** | **10.** |
| --- | --- | --- | --- | --- | --- | --- | --- | --- | --- | --- |
| 1. Bacteriologically diagnosed | - |  |  |  |  |  |  |  |  |  |
| 2. Testing rate | -0.06 | - |  |  |  |  |  |  |  |  |
| 3. Vaccination coverage | -0.11 | 0.18 | - |  |  |  |  |  |  |  |
| 4. Under five mortality rate | 0.07 | 0.23* | -0.10 | - |  |  |  |  |  |  |
| 5. Poverty headcount ratio | 0.35*** | -0.37*** | -0.76*** | 0.13 | - |  |  |  |  |  |
| 6. Migrant distribution | -0.32*** | 0.59*** | 0.66*** | 0.20* | -0.62*** | - |  |  |  |  |
| 7. Facility density | 0.05 | 0.11 | 0.48*** | -0.01 | -0.45*** | 0.60*** | - |  |  |  |
| 8. Treatment success rate | -0.19* | 0.03 | 0.09 | 0.00 | -0.05 | 0.17 | -0.09 | - |  |  |
| 9. Test positivity rate | 0.12 | -0.24** | 0.02 | -0.52*** | -0.07 | -0.16 | 0.17* | -0.08 | - |  |
| 10. Literacy rate | -0.23* | 0.21* | 0.70*** | -0.08 | -0.86*** | 0.53*** | 0.39*** | 0.03 | -0.02 | - |
| * p<0.05, ** p<0.01, ***p<0.001. |  |  |  |  |  |  |  |  |  |  |
